# Mucocutaneous manifestations of Behçet’s disease: Pathogenesis and management from perspectives of vasculitis

**DOI:** 10.3389/fmed.2022.987393

**Published:** 2022-12-02

**Authors:** Doyoung Kim, Koichiro Nakamura, Fumio Kaneko, Erkan Alpsoy, Dongsik Bang

**Affiliations:** ^1^Department of Dermatology, Cutaneous Biology Research Institute, Yonsei University College of Medicine, Seoul, South Korea; ^2^Department of Dermatology, Saitama Medical University, Saitama, Japan; ^3^Institute of Dermato-Immunology and Allergy, Southern Tohoku General Hospital, Fukushima, Japan; ^4^Department of Dermatology and Venereology, School of Medicine, Akdeniz University, Antalya, Turkey; ^5^Department of Dermatology, Catholic Kwandong University, International St. Mary’s Hospital, Incheon, South Korea

**Keywords:** Behçet’s disease, vasculitis, mucocutaneous, pathogenesis, thrombosis

## Abstract

Behçet’s disease (BD) is a systemic inflammatory disorder characterized by vasculitis affecting blood vessels of any caliber or type. It can present with a wide spectrum of vasculitic lesions, including erythema nodosum-like lesions and retinal vasculitis, and may also lead to larger vessel diseases, such as aortic aneurysm and deep vein thrombosis. The full etiology of BD remains unclear, but it is considered a polygenetic disease with multiple genetic risk factors that promote immune dysregulation and thrombophilia. Inflammation can be triggered by environmental factors, such as bacteria or viruses, and the dysregulation of innate and adaptive immune cell subsets. Neutrophils and lymphocytes are the primary players involved in BD pathogenesis, with specific innate (i.e., neutrophil-derived reactive oxygen species and neutrophil extracellular traps) and adaptive (i.e., anti-endothelial cell antibodies) processes inducing endothelial cell activation and chemotaxis of inflammatory cells, leading to coagulation and vasculitis. These inflammation-induced vasculitic or vasculopathic features are observed in most mucocutaneous BD lesions, although vasculitis *per se* is often pathologically evident only during a brief period of the disease process. Due to the multifactorial nature of BD-associated inflammation, broad-spectrum anti-inflammatory medications, including glucocorticoids and immunosuppressive drugs, have been the mainstay for managing BD. In addition, inhibitors of interleukin (IL)-1, tumor necrosis factor (TNF)-α, and IL-17, which target innate and adaptive immune functions dysregulated in BD, have emerged as promising new therapeutics. In this review, we discuss the muco-cutaneous manifestations of BD by focusing on the underlying vasculitic components in their pathologies, as well as the current array of treatment options.

## Introduction

The term vasculitis generally refers to an inflammation within the blood vessel wall, leading to its destruction. In contrast, conditions involving the formation of a thrombus within the vascular lumen that compromises blood flow, as well as more general blood vessel diseases, are known as vasculopathies. Historically, vasculitides—autoimmune diseases characterized by vasculitis—have been classified by the size of the vessel involved. However, one member of this group, known as Behçet’s disease (BD), has been defined as variable vessel vasculitis by the 2012 Revised International Chapel Hill Consensus Conference (1), meaning it can affect vessels of any size (i.e., small, medium, or large) and type (i.e., arteries, veins, or capillaries). For example, many BD patients develop posterior uveitis with severe retinal vasculitis, including typical vascular pathologies with endothelial activation and diffuse capillary leakage. Conversely, in a subset of BD patients, inflammatory vascular damage occurs on larger vessels, and this often presents with life-threatening sequelae, such as an aortic aneurysm or deep vein thrombosis (DVT). Critically, the pathologic cues driving vessel damage in BD remain elusive, and not all clinical manifestations of BD, particularly mucocutaneous lesions, are directly related to vasculitis in their pathology. Herein, we review BD pathogenesis with respect to vasculitis and provide updated clinical information and therapeutic recommendations for mucocutaneous BD, with special emphasis on idiopathic immune-mediated vasculitis.

## Pathogenesis

### General overview of Behçet’s disease pathogenesis

Behçet’s disease is a systemic inflammatory disease characterized by recurrent oral and genital ulcerations, inflammatory skin lesions, and uveitis. Various systemic manifestations, including arthritis, as well as gastrointestinal, neurological, and vascular involvements, can also occur, and life-threatening complications may be accompanied by severe inflammation of internal organs. The etiological mechanisms underlying BD pathogenesis remain to be elucidated, although it is hypothesized to result, in part, from immune dysregulation in genetically susceptible individuals, which is provoked by environmental factors, such as an infectious agent or trauma. Consequently, genetic predisposition, the role of environmental factors, and innate/adaptive immunological consequences have been widely studied in the context of BD (2, 3).

#### Role of genetic factors

Previous studies have found that the HLA-B51 allele of the major histocompatibility complex (MHC) is strongly associated with BD development across all ethnicities. In particular, a meta-analysis that included data from 78 independent studies and 4,800 BD patients reported that the odds ratio (OR) of BD development in those with the HLA-B5/B51 allele vs. those without this allele was 5.78 (95% confidence interval: 5.00–6.67) (4). However, it seems unlikely that one specific HLA allele can fully explain the pathogenesis of BD. Accordingly, genome-wide association studies (GWAS) have further contributed to our understanding of BD pathogenesis by uncovering novel susceptibility genes. A recent GWAS, for example, identified a genetic interaction between HLA-B*51 and the endoplasmic reticulum aminopeptidase 1 (*ERAP1*) gene in BD (5). As its name suggests, the product of this gene is an endoplasmic reticulum-expressed aminopeptidase that trims antigen peptides to an optimal size before loading onto MHC class I molecules. Intriguingly, the observed genetic epistasis in which homozygosity for *ERAP1* p.Arg725Gln mutation strongly increases the OR for BD development in HLA-B51 + vs. HLA-B51- individuals, suggests a role for MHC-I, peptide, and T cell interactions in BD pathogenesis, thereby revealing a possible MHC-I-opathy (6). However, the severe phenotype of BD is not uncommon in patients lacking HLA-B51; thus, the causal role of MHC in BD should not be overrated (7).

In addition to HLA and *ERAP1*, a series of GWAS further identified BD susceptibility loci at multiple genes related to innate and adaptive immune function. In one case, polymorphisms on interleukin (*IL)23R/IL12RB2* and *IL10* loci were found to be closely correlated with dysregulation of inflammatory cytokine profiles in BD patients (8). Levels of both T helper (Th)1- and Th17-related cytokines such as IL-12, interferon (IFN)-γ, IL-17A, IL-17F, IL-22, and IL-23 have also been widely investigated in serum, blood immune cells and tissues of active BD patients (9). These studies suggest genetic variants in the adaptive immune system are directly related with immunophenotype of BD. Notably, the *IL10* variants associated with BD-susceptibility generate a reduced expression of this anti-inflammatory cytokine, which may lead to an imbalance between pro-inflammatory processes and immune regulation (8). In addition, many other susceptibility loci for BD development are also located in genes related to the innate immune system, including C–C motif chemokine receptor (*CCR)1-CCR3*, killer cell lectin-like receptor C4 (*KLRC4*), *IL1B*, interferon regulatory factor (*IRF)8* and interferon gamma receptor 1 (*IFNGR1*) (5, 10, 11). Thus, a genetic predisposition for BD is associated with alterations in both adaptive and innate immune system function, which correspond to the clinical spectrum of BD characteristics, more broadly, to the manifestations of numerous autoimmune diseases and autoinflammatory syndromes.

#### Role of environmental factors

Infectious agents have long been proposed as triggering factors for BD development. In particular, many studies suggest that a cross-reactive immune response against human proteins possessing high homologies with certain antigens from bacteria (i.e., *Streptococcus sanguinis*) or viruses [i.e., herpes simplex virus (HSV)-1] plays a key role in BD pathogenesis (12). Results from one previous study revealed that the product of the Bes-1 gene and heat shock protein (HSP)-65 derived from an uncommon serotype of oral *S. sanguinis* show high degrees of sequence similarity to the retinal protein BRN3b and human HSP60, respectively (13). Clinically, pathergy tests with self-saliva were found to elicit an increased prevalence of positive reaction compared with control saline, suggesting that hypersensitivity to oral streptococci may induce an innate immune response contributing to BD pathology (14). In addition, the observation of distinct Th1 cell responses in peripheral blood mononuclear cells (PBMCs) derived from HLA-B51 + carriers and non-carriers upon challenge with *S. sanguinis* antigens suggests that antibacterial T cell-mediated immune responses may be at least somewhat dependent on genetic predisposition (15).

Studies have also suggested a viral etiology for BD by reporting the detection of greater quantities of HSV-1 DNA in saliva, genital ulcers (GUs), intestinal ulcers, and PBMCs from BD patients (16, 17). Additionally, using an *in vitro* model with cultured human dermal microvascular endothelial cells, HSV-1 was found to increase expression of cell adhesion molecules, such as intracellular adhesion molecule 1 (ICAM1/CD54), vascular adhesion molecule 1 (VCAM1), and E-selectin on endothelial cells, resulting in increased binding of immune cells to the endothelium (18). Moreover, repetitive inoculation of HSV-1 on the scratched earlobe of the Institute of Cancer Research (ICR) mice induces BD-like symptoms, including skin and GUs, eye symptoms, arthritis, and gastrointestinal ulcers (19). Intriguingly, in addition to HSV-1, the housing environment and fecal microbiota were also important factors for eliciting an inflammatory phenotype in this induced mouse model (20). Given the clinical ineffectiveness of anti-viral agents alone for treating active BD patients (21), this suggests that rather than a direct role for HSV-1 infection in BD, HSV-1-induced immune dysregulation may contribute to the induction of BD pathology in conjunction with immune responses to other environmental factors, including bacteria. Recent epidemiological trends showing a decreasing incidence of BD in developed countries further support the importance of infectious agents in triggering BD pathogenesis (22–24).

#### Immunological dysregulation

##### Innate immune system

Neutrophil hyperreactivity is a key feature of BD pathogenesis, with neutrophils from active BD patients showing a higher migratory capacity (25) and exhausted phagocytic activity (26), relative to those from healthy controls. Neutrophil infiltration and release of reactive oxidative species (ROS) from these cells also contribute to tissue injury, inflammation, and thrombosis (27), and the release of neutrophil extracellular traps (NETs) may further augment these processes (28). Of note, neutrophils from BD patients exhibit spontaneous NETosis compared with those from healthy controls, suggesting that targeting NETosis might represent a promising therapeutic approach for preventing BD-associated thrombosis and vascular events (29).

Various other innate immune cell subsets, including monocytes, natural killer (NK) cells, and γδ-T cells, are also thought to contribute to BD pathogenesis. Notably, inflammatory monocytes are increased in BD, and these cells express higher levels of TLR2 and TLR4, both of which contain genetic susceptibility loci for BD development (30, 31). Another GWAS-identified locus, which is associated with decreased expression of CCR1 and increased expression of CCR3, is also related to monocytes (5), as functionally, this allele corresponds to their polarization into inflammatory M1 macrophages over regulatory M2 macrophages (32). Moreover, the *IFNGR1* gene has been recently identified as a susceptibility locus for BD in a large multi-ethnic GWAS, and functionally, BD-risk variants show increased expression of IFNGR1 on monocytes (10). Taken together, these findings suggest that certain cellular subsets involved in innate immunity, including neutrophils and monocytes, are closely associated with a genetic predisposition for BD development *via* their critical roles in microbial sensing, thrombogenesis, and fine-tuning of the adaptive immune system.

##### Adaptive immune system

The adaptive immune system, including both Th1- and Th17-mediated immune responses and related cytokines, also plays an important role in the pathogenesis of BD. Upon IL-12 or IL-23 stimulation, naïve CD4 + T cells can differentiate into two cell types: (1) Th1 cell subsets, which secrete IFN-γ, IL-2, and TNF; and (2) Th17 cells expressing IL-17A/17F and IL-22. Of these, the levels of IL-12 and IFN-γ are significantly increased in the blood of BD patients (33), and IFN-γ is increased in the aqueous humor of patients with BD uveitis (34). Similarly, a higher percentage of Th17 cells are present in blood from individuals with active BD, and serum levels of Th17-related cytokines, including IL-17 and IL-23, are increased in BD patients (35, 36). In contrast, regulatory T cells are suppressed in active BD in a Th17-derived IL-21-mediated manner (37). These data, combined with the identification of disease susceptibility loci in *IL23R-IL12RB2, IL10, STAT4*, and *IFNGR1*, as noted above, further support a direct role for a Th1- and Th17-skewed adaptive immune response in BD pathogenesis (5, 8, 10). As monocytes from BD patients facilitate Th1 and Th17 differentiation of T cells in an allogeneic co-culture model (38), it is likely that innate players, including antigen-presenting cells (APCs), are involved in shaping the dysregulated adaptive immune response present in BD.

### Vasculitis and thrombosis in Behçet’s disease

Although the precise mechanisms remain unclear, it is thought that the inflammatory processes outlined above induce activation of the vascular endothelium *via* cytokine signaling, resulting in BD-associated vasculitis. This condition has a number of key features suggesting the close connection between inflammation, endothelial damage, and thrombogenicity. Under normal physiological conditions, activation of the coagulation cascade by inflammation is part of a natural defense mechanism against pathogens. However, aberrant inflammation can induce thrombosis, which in turn, amplifies inflammation, leading to so-called immuno-thrombosis (39). This process may further lead to the recruitment and activation of neutrophils and other immune cells, which are partially modulated by the endothelium.

The early observation of polymorphonuclear neutrophils adhering to endothelial cells and their subsequent migration into inflamed areas supports the importance of enhanced leukocyte chemotaxis and the critical role of vasculopathy in BD pathogenesis (40, 41). It was subsequently shown that excessive levels of ROS produced by neutrophils modify the structure of fibrinogen, generating an altered architecture that is less susceptible to plasmin-induced lysis (27). In addition, as noted above, neutrophil-produced NETs are also associated with thrombophilia in BD (29). Collectively, these data are consistent with early ultrastructural observations suggesting that endothelial cell damage and subsequent necrosis of damaged cells are the initial events leading to thrombosis in BD skin lesions (42, 43). Radiological observation of thickened vessel walls in patients with, or even without, vascular BD, when compared to vessel walls (i.e., femoral vein) in healthy controls, further supports the hypothesis that endothelial activation, not thrombosis, is the primary event in this disease (44, 45).

**FIGURE 1:**
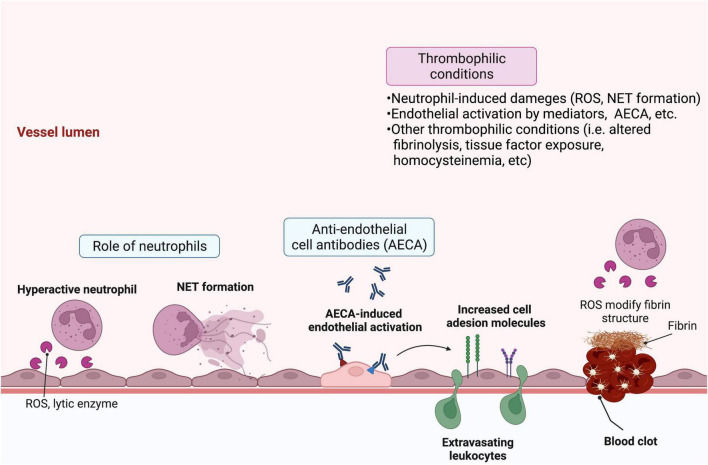
Schematic overview of the pathologic features of Behçet’s disease (BD) that lead to vasculitis and thrombosis. Various factors contribute to endothelial damage and subsequent vasculitis and thrombosis in BD. Hyperactive infiltrating neutrophils release reactive oxygen species (ROS) and lytic enzymes and may form neutrophil extracellular traps (NETs), which activate or damage endothelial cells. Circulating anti-endothelial cell antibodies specific for various endothelial target proteins can further activate endothelial cells. In turn, they upregulate cell adhesion molecules that stimulate leukocyte migration. Moreover, activated endothelial cells can secrete proteins, including plasminogen activator inhibitor-1 (PAI-1) and tissue factor, which contribute to clotting and thrombosis. Patient characteristics, such as high levels of plasma homocysteine or neutrophil-derived ROS, can further augment thrombophilic activity *via* dysregulation of fibrinolysis. Created with BioRender.com.

A different approach for investigating the factors that initiate endothelial damage in BD identified serum anti-endothelial antibodies as a key trigger. Specifically, using endothelial cells from human umbilical veins and adipose tissue, Cervera et al. detected an increased level of serum anti-endothelial antibodies in BD patients, which are correlated with disease severity (46). These anti-endothelial cell antibodies induce increased expression of cell adhesion molecules on endothelial cells (47), a phenotype that is functionally associated with neutrophil recruitment (48). Subsequent proteomics analyses identified target proteins for the different isotypes of anti-endothelial antibodies, including IgM anti-human α-enolase and IgA anti-heterogeneous nuclear ribonucleoprotein (hnRNP) A2/B1 antibodies, which cross-react with streptococcal antigens (49, 50). Subsequently, additional proteins, including prohibitin (51), HSP27 (52), and annexin A2 (53), were identified as targets for anti-endothelial cell antibodies. Given the strong association between levels of anti-hnRNP A1 IgG anti-endothelial antibodies and DVT observed in a large cohort study, it is likely that the autoimmune mechanism of BD is closely linked to vascular involvement and thrombotic tendency (54). However, the specific question regarding precisely how endothelial damage can induce thrombotic tendency in BD remains unanswered. Overall, the data suggest a model whereby increased oxidative stress at sites of inflammation resulting from neutrophil recruitment and activation likely contributes to endothelial cell damage, and the production of anti-endothelial antibodies against various antigens exposed from endothelial cells further stimulates endothelial activation *via* molecular mimicry. In addition, subsequent dysregulation in thrombogenesis and fibrinolysis, subsequently, a multifactorial process related to fibrinolysis including secretion of endothelial-derived proteins [i.e., plasminogen activator inhibitor 1 (PAI1)], tissue factor exposure, and inherent dysregulation in plasma homocysteine levels may contribute together to vascular involvement in BD (Figure 1) (55, 56).

Behçet’s disease partly shares clinical and pathogenic features with other types of primary vasculitides. Anti-neutrophil cytoplasmic antibody (ANCA)-associated-vasculitis (AAV), a representative vasculitis group involving small sized vessels, also manifests various clinical symptoms and signs related to a systemic inflammatory response, end organ microvascular injury, or the mass effect of granulomas. Despite heterogeneity among subgroups of AAV, both arterial events and venous thrombosis occurs frequently in AAV. Furthermore, genetic risk alleles for the development of AAV include HLA SNPs (i.e., HLA-DP, HLA-DQ), innate (i.e., *TLR9*) or adaptive (i.e., *CTLA4, FCGR3B, IL10, IL2RA*) immune response, and signal transduction (i.e., *PTPN22*) (57). However, characteristic predilections for organ involvement in AAV subgroup exist and the presence of ANCA directed to proteinase 3 (PR3-ANCA) or myeloperoxidase (MPO-ANCA) can be differentiated from BD. Of note, genes encoding ANCA associated proteins such as proteinase 3, serpin family A member 1 are associated with disease development, and the direct role of ANCA binding to Fcγ receptor on neutrophils in NET formation and endothelial damages is more clearly defined (58–60). In summary, comparison between BD and other type of primary vasculitides highlights both similar features as immune-mediated vasculitis/thrombophilia and disease-specific dissimilarities in genetic and immune pathogenesis between conditions.

## Clinical features

Diagnosis of BD is primarily based on clinical symptoms, as there are no diagnostic laboratory findings. Oral ulcers (OUs), GUs, skin lesions, and uveitis comprise the major Japanese diagnostic criteria (61), whereas the International Study Group (ISG) criteria include OUs, GUs, uveitis, skin lesions, and positive pathergy test (62). The International Criteria for Behçet’s Disease (ICBD) criteria also include a positive pathergy test with the four major symptoms noted above, as well as the presence of neurologic and vascular lesions (63).

### Mucocutaneous manifestations

Mucocutaneous lesions, which are included in all the above-mentioned diagnostic criteria, are the most common symptom of BD at onset or at any stage of the disease and persist with recurrent attacks throughout the disease course. These may include erythema nodosum (EN)-like lesions, papulopustular lesions (PPLs), superficial thrombophlebitis, and pathergy reactions. Notably, the appearance of mucocutaneous lesions precedes by several years the onset of internal organ involvement, such as the development of ocular or vascular lesions, suggesting that these represent a key manifestation of early BD pathogenesis (64).

#### Oral ulcers

Oral ulcers often appear as the first disease manifestation and are present in most patients with BD. These can develop on the lips, gingiva, buccal mucosa, and tongue and resemble recurrent aphthous stomatitis (RAS). BD-associated OUs can be classified as minor, major, or herpetiform, depending on their size and number. Minor OUs are the most common (80–85% of cases) and are distinguished by small (<1 cm), shallow ulcers that heal within 1–2 weeks without scarring. Major OUs are less commonly seen (10–15% of cases); they are morphologically similar to minor OUs but are larger (>1 cm), deeper, and more painful. Major OUs also last longer than minor OUs and frequently heal with scarring and tissue loss. Herpetiform OUs are the rarest form (5% of cases); these are 1–3 mm in size and 10–100 in number (65). Notably, the presence of major OUs is a characteristic of BD that allows it to be clinically differentiated from RAS.

The recurrence of OUs in BD is affected by fatigue, stress, food, smoking cessation, and menstruation. In addition, conditions associated with poor oral hygiene, such as periodontitis, chronic tonsilitis, and tooth decay, are often observed in BD patients. Therefore, we have previously proposed that species of oral bacterial flora, particularly *S. sanguinis*, can be triggers for OUs in BD (66). In support of this hypothesis, results from *in vitro* experiments revealed that inflammatory cytokines, including IFN-γ and IL-6, are produced by PBMCs from BD patients in response to stimulation with Streptococcal antigen derived from *S. sanguinis* (67). Titers of serum antibodies against *S. sanguinis* were also found to be elevated in BD patients (68). Further, as noted above, HSP-65 peptides produced by *S. sanguinis* show considerable sequence homology to the human HSP60 protein, and intriguingly, the human HSP60 peptide induced proliferation of T cells in BD patients, but neither in healthy controls nor patients with rheumatoid arthritis (69). Elevated serum levels of HSP60 and VEGF were detected in BD patients, and the serum level of VEGF is correlated with vascular involvement (70). Thus, it is thought to be involved in the generation of vascular inflammation, leading to vascular damage in BD.

#### Genital ulcers

Genital ulcers, which occur in more than 60% of BD patients, are the second-most common manifestation at disease onset after OUs and are also a specific clinical finding for BD diagnosis. In male patients, GUs often occur on the scrotum and penis, whereas in women, GUs are commonly found on the major and minor labia. Large ulcers are deep and sometimes leave a scar. For diagnosis of BD, these painful ulcers should be differentiated from herpes infection, which produces grouped, small, shallow ulcers that recur in the same location. The presence of multinucleated acantholytic cells in the Tzanck smear or HSV-PCR test positivity can differentiate HSV infection from the GUs of BD.

#### Erythema nodosum-like lesions

Erythema nodosum-like lesions are identified in one-third to half of BD patients and are more common in females. These are painful oval-shaped erythematous subcutaneous nodules that frequently involve the pretibial region and are often associated with fever, malaise, and arthritis. EN-like lesions of BD are relatively small and heal within 1–3 weeks without a scar; however, they typically recur over long periods. EN is not specific to BD, and classic EN is often associated with bacterial and viral infections, as well as with conditions such as pharyngitis, Crohn’s disease, ulcerative colitis, and Sweet’s syndrome. Therefore, both clinical and histological differential diagnosis is necessary to distinguish EN-like lesions of BD from classic EN.

#### Papulopustular lesions and acneiform eruption (pseudofolliculitis)

Papulopustular lesions and acneiform eruption in BD are folliculitis- or acne-like sterile pustules on the face, neck, and extremities that rapidly appear and are present in more than 60% of BD patients. They are usually small, uniformly shaped, non-follicular lesions, which heal quickly without scaring but often recur with pain. PPL can be developed either as non-follicular or follicular based on lesion morphology but non-follicular PPL localized in the lower extremities has been reported to be more specific for BD (71).

#### Pathergy

Pathergy, or needle reaction, is a test that measures erythematous papule or pustule formation in response to a prick with a sterile needle, which develop 24–48 h after the test is administered. Positive pathergy is a cutaneous hypersensitivity reaction against trauma and a characteristic feature that occurs more frequently in active BD patients. In the early phase of BD, pathergy is identified at the site of injection and infusion. The positivity rate for needle reaction among BD patients is 50% in the eastern Mediterranean region, such as in Turkey and Iran, but relatively low at less than 30% in Asian countries, such as Korea and Japan. These contrasting results in distinct geographic regions are attributed to differences in pathergy test application methodology and ethnic characteristics (65). It has been suggested that the pathergy reaction might be a response to bacteria residing on the skin surface, although, at present, no clear causation has been confirmed. Notably, we previously showed that needling with autologous oral salivary fluid on it induced a positive pathergy reaction on the forearm of BD patients (14). However, because the patient numbers in this study were small, a definitive conclusion awaits further investigation.

#### Superficial thrombophlebitis

Superficial thrombophlebitis is a cord-like painful induration along the vein in the legs, which also sometimes occurs on the forearm after intravenous injection in those with BD. Importantly, when multiple superficial thrombophlebitis lesions are observed in a BD patient, the individual should be carefully examined for vascular lesions involving the deep veins or major vessels in the internal organs (e.g., pulmonary arterial thrombosis).

### Histopathological features of vasculitis in mucocutaneous symptoms of Behçet’s disease

Vasculitis is the fundamental pathologic characteristic of the BD skin lesions described above, with thrombophlebitis (i.e., thrombus-associated inflammation) representing the second-most important manifestation in mucocutaneous BD lesions. Critically, both of these characteristic features are also present in the major organs of the intestinal, vascular, and central nervous systems that are affected by BD. Because the onset of major internal organ involvement follows the appearance of mucocutaneous symptoms by several years, the observation that key histological features of mucocutaneous lesions precede and predict the appearance of major internal organs affected by BD is an extremely important finding.

Aphthous OUs in BD patients typically contain neutrophilic perivascular infiltration, and both macrophages and phagocytic apoptotic cells can be identified in the damaged epithelial layers, a feature that is not present in normal oral mucosal tissue (72). We have further shown that the epithelial component at the margin of BD-associated OUs is immunostained by anti-human IgA, IgM, complement, and streptococcal antibodies in BD patients (66).

**FIGURE 2:**
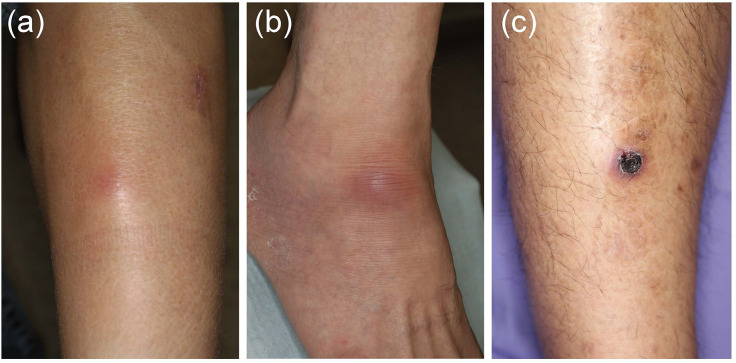
Various skin lesions that may be present on the lower legs in Behçet’s disease (BD) patients. **(a)** Case 1, erythematous subcutaneous nodule on the leg. **(b)** Case 2, erythematous plaque on the ankle. **(c)** Case 3, crusted ulcer on the leg.

**FIGURE 3:**
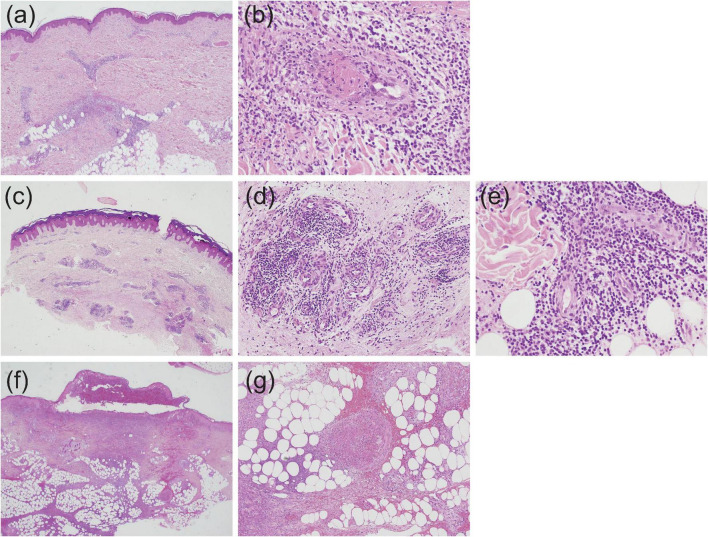
Histopathological findings indicative of vasculitis in Behçet’s disease (BD) skin lesions; all slides were stained by hematoxylin and eosin, and the magnification is shown in parenthesis. **(a,b)** Case 1, perivascular neutrophilic infiltration in the dermis (**a**, ×20) and subsequent occlusion of the involved blood vessel (**b**, ×400). **(c–e)** Case 2, perivascular dense infiltration in the dermis (**c**, ×20), and prominent accumulation of neutrophils and lymphocytes around blood vessels in the mid (**d**, ×200) and deep (**e**, ×400) dermis. **(f,g)** Case 3, extensive dermal infiltration of neutrophils beneath a crusted lesion (**f**, ×20) and occlusion of venous blood vessels and neutrophilic infiltration in septal regions (**g**, ×100).

In EN-like lesions of BD, septal panniculitis can be histologically identified, with a predominantly neutrophilic infiltration in combination with lymphocytes. The blood vessels also often show prominent infiltration of neutrophils and vascular changes (Figures 2A, 3A,B), and venous thrombosis caused by neutrophil infiltration may also be present in the deep dermis. In some cases, vascular damage that is similar to necrotizing vasculitis has further been reported in EN-like BD lesions (Figures 2B, 3C–E). Moreover, as shown in representative ulcerative lesions with crusts on the legs of BD patients, venous thrombosis may be identified in the deep dermis through fat tissue in cutaneous lesions, and this is indicative of vascular BD (Figures 2C, 3F,G). Importantly, because classic EN-like lesions do not ulcerate, when ulcerating EN-like lesions are observed, vascular BD, as well as necrotizing vasculitis and peripheral blood insufficiency should all be considered (73).

Detection of perivascular neutrophil infiltration in the dermis is an important initial histological finding in BD skin lesions. Histochemical analysis showed enhanced expressions of IL-8 and CCL20 (MIP-3α) in pustular skin lesions from BD patients, and the isolated skin-infiltrated T cells produced high amounts of IL-8 (74). Moreover, the serum level of IL-8 correlates with disease activity, and the serum IL-8 level was elevated in active BD patients with vascular involvements (75). These data suggest that the cytokines and other proinflammatory factors, which activate neutrophils, contribute to the induction of both vasculitis and venous thrombosis. After inflammation, a secondary cause of thrombophilia in BD is thought to be myeloperoxidase, which is produced by active neutrophils and may be associated with endothelial cell abnormalities and induction of vascular damage (48). In addition, as noted above, NETs have been reported with thrombophilia in BD (29).

Papulopustular lesion in Behçet’s disease presents with papules and pustules that are mainly observed on the trunk, lower extremities, upper extremities, and face (76). Of note, similar to OUs and EN-like lesions in BD, vascular-related histological findings have also been reported in BD-associated PPL (77, 78). One study found that out of 42 BD patients with PPL, seven (16.7%) showed histological features of leukocytoclastic vasculitis, and 27 (64.3%) displayed superficial and deep perivascular inflammation and/or interstitial infiltration; no vasculitis was observed in controls with acne vulgaris (77). In a separate study, Chen et al. (79) reported that 20 out of 42 patients (48%) had signs of cutaneous vasculitis, with 17% showing leukocytic vasculitis and 31% displaying lymphocytic vasculitis. Consistent with these observations, on histology, non-follicular PPLs from BD patients were found to contain significantly more leukocytoclastic vasculitis than non-lesional skin, with lesional vessels showing IgM, IgG, C3, and fibrin deposition (71). These features indicate that non-follicular PPLs are characteristic cutaneous manifestations of BD with significant diagnostic value for identifying vasculitis in BD lesions. Interestingly, most BD patients with clinical PPL histologically displaying signs of vasculitis were male (90%), a finding consistent with the natural course of BD, in which severe cases occur more commonly in males than in females.

In superficial thrombophlebitis, occlusion of the lumen within venous blood vessels can be histologically identified in subcutaneous lesions, along with perivascular neutrophilic infiltration. Therefore, this condition shares clinical features with EN-like lesions and vasculitis in the leg, wherein induration is cord-like, and histological examination is necessary in such cases. The ultrastructural examination can identify vascular changes in cutaneous BD lesions (80), and multiple superficial thrombophlebitic lesions are often associated with the existence of DVT. Thus, to examine DVT and differentiate BD from superficial thrombophlebitis, magnetic resonance imaging (MRI), computed tomography (CT), and other imaging techniques are helpful.

## Therapeutic approaches for mucocutaneous Behçet’s disease

### Topical treatment

The European Alliance of Associations for Rheumatology (EULAR) task force has stated that treatment decisions for patients with BD may depend on the severity of mucocutaneous lesions, as well as the dominant or codominant lesions present (81, 82). Topical treatment is generally prescribed as an adjunct to systemic therapy. However, topical measures alone can be administered in cases showing remission for a long period, those without major organ involvement, or elderly patients without severe organ involvement (83). In mild cases of OU, therapeutic approaches may also involve a mild diet and avoiding consumption of spicy, salty, or hard to digest foods, as well as synthetic additives (83).

Topical corticosteroids are efficacious for the treatment of most mucocutaneous lesions. One randomized comparative study showed better efficacy for 0.1% triamcinolone acetonide ointment vs. an active comparator, phenytoin syrup, for the control of OUs in BD (84). Thus, based on the clinical benefit of topical corticosteroids for OUs in RAS, these are recommended as the initial treatment choice for uncomplicated OUs in BD. Further, while the effectiveness of topical corticosteroids for the treatment of GUs and EN-like lesions has not been investigated in randomized clinical trials, they have long been empirically used to treat GUs and are listed as first-line treatment in the EULAR recommendations (82). Topical steroids can also be used to treat EN-like lesions, and the Japanese guideline for mucocutaneous lesions recommends topical steroids for mild-to-moderate cases with EN-like lesions (85). The efficacy of topical steroids for BD-associated PPL is limited. However, PPL is more common in patients with a positive pathergy test, suggesting that topical steroids may hold potential benefits for resolving inflammatory PPL caused by the hypersensitivity reaction in BD (76). In addition to topical corticosteroids, 3 months of topical sucralfate treatment for OUs effectively reduces pain and time to healing (86). Pimecrolimus cream in combination with colchicine also helps to shorten the healing time of GUs in BD (87).

### Systemic treatment

Several systemic treatments may be considered in BD patients with mucocutaneous lesions, depending on the clinical spectrum and severity of the disease (81, 82, 85). They are described in more detail below.

#### Colchicine

Colchicine suppresses neutrophil function and cytokine release, and it is therefore an integral component of first-line treatment for BD patients with lesions in various organs (81, 85). Notably, the effectiveness of colchicine for the treatment of OUs, Gus, EN-like lesions, and arthritis in BD has been demonstrated in a placebo-control trial (88). In contrast, another study did not find significant benefits for colchicine in controlling OUs, and it was found to be effective for GUs and EN-like lesions in female patients but not in males (89). However, this study may have been biased due to the fact that topical therapy, acetaminophen, and NSAIDs were not strictly controlled. Thus, despite inconsistent performance across multiple studies, colchicine can be generally recommended for moderate-to-severe mucocutaneous cases, such as those involving OUs, GUs, EN-like lesions, and PPL (85).

Colchicine has also been reported to inhibit platelet aggregation and prevent thrombosis effectively (90), and a combination of anticoagulant and colchicine was shown to be effective in pediatric BD cases involving venous thrombosis (91). Based on results from these studies, we recommend colchicine for the treatment of moderate-to-severe cases of superficial thrombophlebitis, even though, to date, no randomized trial of colchicine for superficial thrombophlebitis has been performed.

#### Corticosteroids

Systemic corticosteroids rapidly suppress inflammation and cytokine production, and thus, these have been empirically used to control acute and severe attacks of mucosal ulcerations in BD (92). One randomized trial found that intramuscular injection of methylprednisolone acetate was effective for EN-like lesions, but not for GUs (93). Overall, despite the paucity of well-designed studies, we recommend systemic corticosteroids as a treatment option for mucocutaneous lesions in severe or intractable BD cases (85).

#### Apremilast

Apremilast is an oral phosphodiesterase-4 inhibitor that has recently become available for the treatment of inflammatory skin diseases, such as psoriasis. In a randomized trial, apremilast effectively decreased both pain and the total number of OUs after 12 weeks of treatment compared to placebo, and these effects were maintained for up to 64 weeks (94, 95). A recent meta-analysis on eight related trials further verified that apremilast significantly induces symptom-free remission for GUs, EN-like lesions, pseudofolliculitis, and arthritis at 12 weeks (96). However, as the side effects of apremilast include diarrhea, headache, and nausea, the EULAR task force has recommended the use of apremilast only in selected BD cases (81).

#### Mucosal protectants and antimicrobial agents

Rebamipide, a mucosal protectant, has been shown to reduce both the number and pain of BD-associated OU lesions (97). Similarly, sucralfate suspension, most commonly used for treating duodenal ulcers, was also found to improve oro-genital BD ulcers. Therefore, both of these agents are recommended for treatment of mucosal ulcerations in BD (86). Antimicrobial drugs have also displayed efficacy for BD treatment. For example, benzathine penicillin significantly improved the frequency and duration of OUs when used with colchicine (98). Similarly, various formulations of topical antimicrobial agents, including chlorhexidine gel, penicillin G potassium troches, amlexanox, tetracycline suspension, and doxycycline powder, are effective for RAS and can be used to treat OUs in BD patients (83). Minocycline has both anti-inflammatory and antibacterial activity and was shown to be effective for decreasing symptoms associated with EN-like lesions, although the study sample size was small and the trial design was non-randomized (99). In summary, these data suggest that topical and systemic antimicrobial agents can be used as auxiliary therapeutics to reduce mucocutaneous inflammation in BD.

#### Anticoagulants

Despite the presence of inflammation-related thrombophilia in BD pathophysiology, the efficacy of anticoagulants for BD treatment remains controversial. In particular, no solid evidence supporting the benefit of warfarin for BD thrombophlebitis has been reported (100). Nonetheless, anticoagulants are often administered along with steroids and immunosuppressive drugs to treat vascular changes in BD. One study by Emmi et al. (101) reported no significant difference in the recurrence rate of venous thrombosis in the group of subjects treated with immunosuppressive drugs alone vs. those treated with both immunosuppressives and anticoagulants. In contrast, another study found that the risk of severe post-thrombotic syndrome, a chronic complication of leg vein thrombosis, was increased in BD patients who did not take anticoagulants in combination with immunosuppressants at the onset of thrombosis (102). This suggests a potential benefit for anticoagulants in patients with chronic lower leg vein thrombosis. In this context, the recently updated Japanese guidelines support the addition of warfarin as an option along with steroids or immunosuppressive agents in clearly indicated situations (85). However, answering the question of whether the addition of anticoagulants to BD therapeutic regimens is effective for treating venous thrombosis will require further investigation in clinical trials, particularly those focused on the use of novel oral anticoagulants (103).

#### Immunosuppressants and immunomodulatory agents

Given the role of inflammation in disease pathogenesis, several immunosuppressant agents have been utilized for BD treatment. For example, azathioprine effectively decreased the number of OUs and GUs in BD patients in a randomized controlled trial (104). Therefore, the EULAR task force recommends azathioprine use in selected cases with mucocutaneous lesions. Additionally, in a small cohort study, cyclosporine A showed clinical efficacy for treating GUs, skin lesions, and superficial thrombophlebitis (105). However, given the potential risk for the development of neuro-BD, the use of cyclosporine A should be reserved for selected cases.

Dapsone (diamino-diphenyl sulfone) inhibits the activation of neutrophils and is widely used to treat of inflammatory skin diseases. In a double-blind controlled study, a decreased number of OUs, GUs, EN-like lesions, and PPL was observed in dapsone-treated BD patients (106). Thus, dapsone can be used as an alternative immunomodulatory drug in refractory cases with mucocutaneous symptoms. In addition, thalidomide has been reported to induce long-term remission of OUs, GUs, and PPL in BD patients; however, this agent should be used only in selected cases due to its potentially severe side effects (107).

#### Tumor necrosis factor-α inhibitors

Inhibitors of the cytokine TNF-α are used to treat various inflammatory diseases, including BD. In particular, a randomized trial and some case reports have provided convincing evidence favoring the use of TNF-α inhibitors for OU treatment. Similarly, the efficacy of TNF-α inhibitors for treating GUs was demonstrated in multiple case reports, and etanercept was found to be beneficial for EN-like lesions in a randomized clinical trial (108). However, given the potentially severe side effects, TNF-α inhibitors can be considered only in cases of severe and intractable mucocutaneous lesions (109).

Tumor necrosis factor-α inhibitors have also been investigated for the treatment of BD-associated thrombosis. One cohort study on BD patients with DVT and/or superficial vein thrombosis found that the adalimumab-based treatment groups (i.e., adalimumab administered alone or in combination with immunosuppressive agents) showed rapid clinical and ultrasonographic improvement compared with those receiving immunosuppressive agents only during a mean follow-up of 26 months (110). In addition, a steroid-sparing effect was observed in the adalimumab-based groups. Thus, we suggest that TNF-α inhibitors, alone or in combination with immunosuppressive agents, can be an option for severe cases of superficial thrombophlebitis or DVT, although further studies are needed.

#### Other biologic therapies

Among the various cytokine therapies available, the efficacy of IFN-α has been widely verified for the treatment of mucocutaneous BD. A placebo-controlled study showed that IFN-α was effective in reducing the healing time and pain of OUs, as well as the frequency of GUs and PPLs (111). In addition, a systematic review examining the use of anti-IL-1 antibodies has shown beneficial effects of anakinra and canakinumab for controlling mucocutaneous lesions of BD (112). Similarly, both ustekinumab, an anti-IL-12 and anti-IL-23 antibody (113), and the anti-IL-17 antibody secukinumab, were found to be effective for treating refractory mucocutaneous BD lesions (114). Thus, these biologics can be tried in patients with intractable cases of mucocutaneous BD.

#### Disease specificity in drug selection

As mentioned above, various therapeutic options exist for the management of mucocutaneous BD. However, it is still not clear how essential the vasculitis itself in clinical presentation of mucocutaneous symptoms. Actually, there is a practical difference in the management compared to other primary vasculitides. The treatment options for other types of primary vasculitides, such as AAV, include cyclophosphamide, mycophenolate mofetil, plasma exchange, and rituximab, which are rarely tried in BD management. On the contrary, apremilast is indicated for the treatment of plaque psoriasis and active psoriatic arthritis but not in primary vasculitis other than BD. Moreover, the favorable efficacy of TNF-α inhibitors as glucocorticoid-sparing agents is shown in managing large vessel vasculitis such as Takayasu’s arteritis, not in most small vessel vasculitis (115). The fact that the treatment option for BD only partially overlaps with other vasculitides suggests a disease-specific aspect of BD pathogenesis and not all treatments directly target the core process of vasculitis itself.

## Conclusion

Behçet’s disease, a systemic vasculitis affecting blood vessels of any caliber or type, is a polygenetic disease associated with multiple genetic risk factors. Inflammation in BD is thought to be triggered by environmental factors, such as microbes or trauma, in genetically susceptible individuals, and both innate and adaptive immune cell subsets, including neutrophils and T cells, are the primary players involved in BD pathogenesis. Histopathological analysis of BD tissue has shown that neutrophils and lymphocytes infiltrate blood vasculatures. This results in vascular endothelial dysfunction and neutrophil-mediated vascular inflammation, which are the key factors inducing thrombophilic features in patients with BD. However, it has been challenging to accurately assess the initial pathologic changes that occur during mucocutaneous lesion formation due to the short-living properties of acute inflammatory cells, such as neutrophils. Therefore, it is still debatable how pivotal the vascular inflammation plays role in the pathogenesis of BD skin lesions despite all the research efforts so far.

Based on the inflammatory origin of BD, broad-spectrum anti-inflammatory medications, including glucocorticoids and immunosuppressive drugs, are the mainstay for managing BD inflammation. In addition, drugs that target dysregulated innate and adaptive immune responses, such as TNF-α and IL-17 inhibitors, have emerged as promising new therapeutics for this disease. However, we are acutely aware that due to the heterogeneity and complexity of this condition, a magic bullet treatment to cure BD is unlikely to be found. Therefore, accumulating an efficacious armamentarium of treatments for BD patient care through the relentless development and verification of diverse therapeutics will continue to be the mission of BD researchers.

## Author contributions

DK, KN, and DB conceptualized and determined the scope for the review. DK and KN drafted the manuscript. DB, FK, and EA were involved in wrote the manuscript and/or revising it critically for intellectual content. All authors contributed to the article and approved the submitted version.
